# Microbial niches at the One Health interface: linking environmental factors, biomes, and disease dynamics

**DOI:** 10.3389/fmicb.2026.1800305

**Published:** 2026-09-11

**Authors:** Aliyu Dabai Ibrahim, Umar Balarabe Ibrahim, Nafi'u Abdulkadir, Shamsudeen Umar Dandare, Juluri R. Rao

**Affiliations:** 1 Agri-Environment Branch, Environment and Marine Sciences Division, Agri-Food and Biosciences Institute Belfast, Belfast, United Kingdom; 2 Department of Microbiology, Usmanu Danfodiyo University, Sokoto, Nigeria; 3 Department of Plankton and Microbial Ecology, Leibniz Institute of Freshwater Ecology and Inland Fisheries, Stechlin, Germany; 4 Department of Microbiology, Sokoto State University, Sokoto, Nigeria; 5 School of Biological Sciences, Queens University Belfast, Belfast, United Kingdom; 6 Department of Biochemistry and Molecular Biology, Usmanu Danfodiyo University, Sokoto, Nigeria

**Keywords:** microbial niches, One Health, human-animal-environment interface, antimicrobial resistance, metagenomics, genome-resolved metagenomics (MAGs), zoonotic pathogen transmission, disease surveillance

## Abstract

Microbial communities shape life at every stratum of environmental hierarchy, ranging from a wide spectrum of functionalities such as nutrient cycling processes that support ecosystem functioning in soil biome to those of intricate balance between disease causal and responsive immune defenses in animals. This review explores the roles of microbial niches in shaping pathogen persistence, antimicrobial resistance (AMR) dissemination, and host–microbe interactions at the human–animal–environment interface. Emphasis is placed on microbial community assembly, environmental drivers of pathogen survival, zoonotic reservoirs, and the contribution of metagenomics and genome-resolved approaches, including metagenome-assembled genomes (MAGs). We examine how microbial diversity is structured by environmental factors, host behaviors, and microbial interactions, with particular attention to the conditions that allow pathogens and antimicrobial resistance genes to emerge or persist. The review highlights how abiotic and biotic factors regulate microbial succession and disease spread across interconnected ecosystems. Literature used in this review was retrieved through a comprehensive search of peer-reviewed publications using major scientific databases using relevant search terms. Findings from this review demonstrate that microbial niches are not passive habitats but dynamic systems that regulate microbial adaptation, pathogen invasion, and ecosystem resilience. Recent advances in metagenomics, genome-resolved metagenomics, and MAG reconstruction and multi-omics technologies have improved the detection and characterization of unculturable microorganisms, antimicrobial resistance genes, and complex microbial interactions. Nevertheless, important knowledge gaps remain regarding how microbial communities respond to environmental pressures and how these responses influence pathogen persistence and antimicrobial resistance dissemination. Therefore, sustained investment in interdisciplinary One Health and MAG-based research, especially in under-resourced regions, will assist in building a resilient and equitable preparedness against emerging infectious diseases and AMR threats.

## 1 Introduction

### 1.1 Historical perspectives of the discovery of microbes, diagnostics, their hierarchy in environments, biome and relevance to One Health

Earlier studies have exhaustively unearthed the “time capsule” of the historical footprints of discovery, definition, description and diagnostic perspectives of the microbe and its world (Millar et al., 2006; Alagona et al., 2020). For instance, in 1676, Antonie van Leeuwenhoek, a Dutch cloth merchant and amateur lens grinder, first observed living microorganisms using his simple microscope, which he called “animalcules”. He examined “animalcules” in the environment, including pond water, sick people, and even his own mouth, and found that these “animalcules” existed everywhere. Over the past four centuries, our understanding of microorganisms has evolved considerably, revealing their roles as both beneficial components of ecosystems and causative agents of disease.

Roman physician Girolamo Fracastoro gave the earliest suggestion that “disease” was caused by invisible living creatures in 1546. Medical professionals and common people did not clearly recognize the role of microorganisms in diseases until 1876, 200 years after Antonie van Leeuwenhoek found his little “animalcules.” Robert Koch established his famous “Koch's postulates” according to the relationship between *Bacillus anthracis* and anthrax, a deadly soil bacterium that affects wild and domestic animals (Logue et al., 2017). Koch was a great pioneer in medical microbiology, and his postulates are still considered fundamental to the field that has continued to evolve via identification and characterization of microorganisms through both conventional and molecular approaches. Comparing century-long conventional detection with molecular detection methods, it is safe to say that the latter have rapidly evolved and are concomitantly being harnessed for the detection of a wide range of microbes in agriculture, veterinary, and human health (Rao et al., 2006) as well as emerging new tools in molecular approaches to soil, rhizosphere, and plant-host beneficial microorganisms (Cooper and Rao, 2006). This includes the early development of techniques based on genomic analysis of soil microbial processes, including metagenomics and the characterization of microbial community structures, which have significantly advanced our understanding of environmental microbiology (Steele and Streit, 2006). The concept of One Health itself has evolved in recent times, with human health widely regarded as not isolated but connected to animal, plants, soil, and water environments (Logue et al., 2017). Microorganisms are now recognized as being shared across different environmental compartments, demonstrating that soil, plant, and human microbiomes are more interconnected than previously thought (Banerjee and van der Heijden, 2022).

### 1.2 The “microbial niche”

A microbial niche refers to the specific ecological role and environment occupied by a microorganism within a given ecosystem (Baquero et al., 2021). This concept encompasses the physical, chemical, and biological conditions that determine where a microorganism thrives and how it interacts with other organisms and its surroundings. The microbial niche is shaped by factors such as nutrient availability, temperature, pH, oxygen levels, moisture, and the presence of symbiotic or competitive organisms (Baquero et al., 2021).

Because of its long history, the term “niche” has been used in a variety of contexts, necessitating clarification of the specific niche concept being considered. A distinction is often made between the fundamental environmental niche and the realized ecological niche. The fundamental niche refers to the set of environmental conditions under which a species can theoretically (i.e., physiologically) survive and reproduce. In contrast, the realized niche represents the subset of those conditions that a species occupies *in-situ* when biological interactions, such as competition and predation, are also fully considered. Thus, the realized niche is a subset of the fundamental niche. Several studies have proposed these two key definitions of the niche and provided frameworks for quantifying niches in the context of species interactions (Remeš and Harmáčková, 2025).

Microbial niches provide the conditions necessary for microorganisms, including pathogens, to survive, proliferate, and interact within ecosystems. The composition and persistence of microbial populations within these niches are strongly influenced by environmental conditions, which shape microbial growth, adaptation, and community dynamics (Nguyen et al., 2021; Baquero et al., 2021). Biotic factors include interactions with other organisms, such as mutualism, competition, and predation, which further regulate microbial distribution and abundance (Faust and Raes, 2012). In addition, the ability of microorganisms to reach and establish in suitable habitats depends on dispersal processes and geographic barriers. Together, these migration-related processes determine with extent to which microorganisms can colonize and occupy available ecological niches (Custer et al., 2022).

### 1.3 Biotic–abiotic–migration (BAM) framework and microbial community interactions

**Figure 1:**
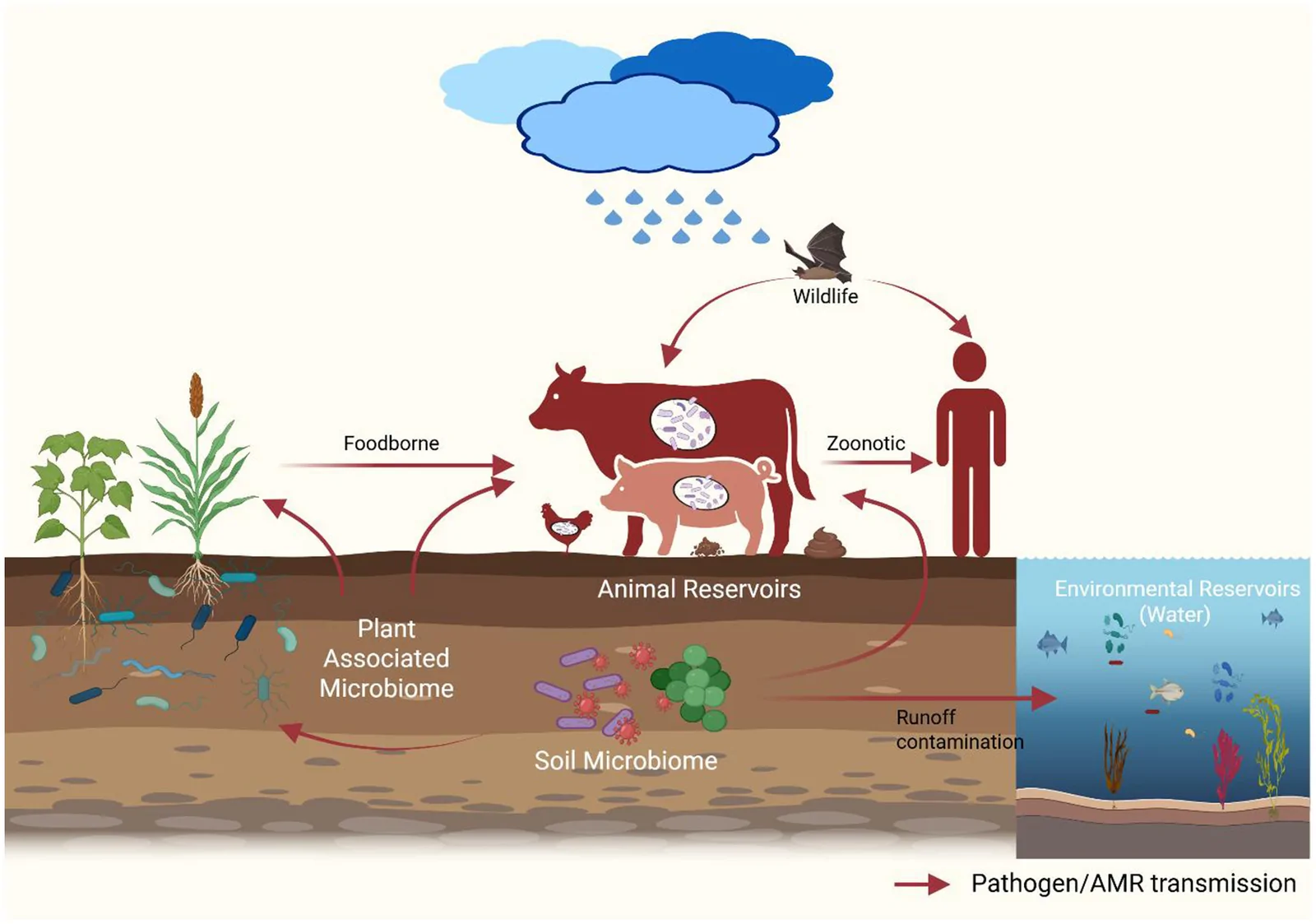
Overview of microbial niches across the One Health interface, illustrating pathogen and antimicrobial resistance (AMR) transmission pathways linking soil, plant-associated microbiomes, animal and wildlife reservoirs, human exposure, and aquatic environmental compartments. Key routes include foodborne transmission, zoonotic spillover, fecal shedding, and runoff-driven contamination. Created in Biorender. Dandare, S. (2026) https://BioRender.com/unkhjts.

Biotic, abiotic and migration are three interrelated sets of factors that are integrated within a framework coded as Biotic–Abiotic–Migration (BAM) framework as reported by Soberón and Peterson (2005). The overlap among these three components corresponds to the realized environmental niche, representing the actual conditions occupied by a species within an ecosystem. Advances in high-throughput amplicon sequencing have provided compelling evidence that microorganisms exhibit distinct habitat preferences, despite the apparent niche differentiation among microorganisms with different functions and across diverse biomes, ecosystems, and hosts (Ni et al., 2025). Distinct taxonomic distributions have been reported across all ecosystems and microbial domains, indicating that different microorganisms occupy discrete environmental niches. An overview of microbial niches and transmission pathways across the One Health interface is presented in Figure 1.

Further classification of niches into trophic, spatial, and functional categories has enhanced our understanding of ecosystem organization based on feeding strategies, energy sources, and nutrient utilization patterns (Kearney et al., 2010). Microorganisms occupy distinct trophic niches depending on how they acquire carbon, energy, and electrons for growth (Steinert et al., 2025). These trophic interactions drive nutrient cycling, maintain ecological balance, and shape microbial community structure across diverse environments.

Microorganisms adapt to their ecological niches through specialized metabolic pathways and genetic modifications (Feng et al., 2025). For example, extremophiles, such as thermophiles and acidophiles, thrive under extreme environmental conditions by producing heat-stable enzymes and acid-resistant proteins (Zheng et al., 2025). Likewise, obligate anaerobes are adapted to oxygen-free environments through fermentation or anaerobic respiration. Microbial communities play a vital role in regulating biogeochemical cycles and sustaining planetary health and productivity by integrating a wide range of metabolic functions across multiple species. These communities consist of diverse microorganisms linked through metabolite exchange, whereby the metabolic by-products of one species serve as resources for another.

Despite their ecological importance and widespread distribution, the fundamental principles governing how microbial communities assemble, collectively process resources, and respond to environmental fluctuations remain poorly understood. This knowledge gap presents a major challenge in microbial ecology, making it difficult to distinguish universal ecological principles from environment-specific patterns.

Although advances in molecular techniques over the past two decades have generated extensive datasets, they have not yet produced a comprehensive theoretical framework for understanding microbial community dynamics (Lahlali et al., 2021). Current research largely relies on genetic markers (e.g., 16S rRNA genes), physiological studies of individual species conducted outside their natural environments, and bulk measurements of chemical inputs and outputs such as O_2_, CO_2_, and ${\text{NO}}_{3}^{-}$ (Beaudry et al., 2021). More recently, thermogravimetric analysis (TGA) has been evaluated alongside conventional PCR-based methods as a rapid detection tool for rhizobacterial biostimulants used in precision agriculture (Carmichael and Rao, 2021).

In soil research laboratories where, microbiological analyses are not routinely performed, thermogravimetric analysis (TGA) has shown potential as a rapid and sensitive tool for detecting chemical signatures associated with plant growth-promoting rhizobacteria (PGPR). Such approaches may support precision agriculture by providing complementary information on microbial inoculant establishment and activity. However, these approaches offer little insight into community dynamics and large-scale genetic data which are difficult to connect to functional processes, as such single-species studies fail to capture interactions within complex microbial communities (Røder et al., 2020). Consequently, linking microbial metabolism to ecological diversity remains a challenge. To uncover the principles of microbial community assembly, we must move beyond merely cataloging species, genes, and metabolites and instead focus on the flows of energy and biomass that drive growth and turnover (Law et al., 2024).

Understanding the relationship between metabolism and community ecology requires studying trophic interactions among microbes in their environments. While the discussion primarily centers on heterotrophic, carbon-limited communities, the concepts presented are also applicable to other systems, including those reliant on inorganic substrates, such as algae–bacteria consortia. In many environments, periodic disturbances create opportunities for microorganisms to colonize new spaces and for microbial communities to reassemble (Philippot et al., 2021). This process can be observed in various contexts, such as the establishment of gut microbiota in new-born animals (Arshad et al., 2021), the colonization of organic matter in the ocean (e.g., decomposing copepods or diatoms; Baumas and Bizic, 2024), and the microbial settlement on plant roots (Wu et al., 2023). Colonization is inherently random, with microbes arriving in different numbers and at different times. Their success depends on both abiotic factors, such as nutrient availability, and biotic interactions with existing microbes, which evolve as the community develops. As conditions shift, microbial populations experience cycles of growth and decline, leading to dynamic successions.

A well-studied example of microbial community assembly occurs in the cow rumen (Pan et al., 2024). At birth, a calf's rumen is colonized by microbes from its mother and environment. Many of these early arrivals cannot survive in the rumen's conditions, but a select few with suitable metabolic traits thrive, shaping the environment through their activity. Initially, oxygen is abundant, but as certain microbes consume it, the rumen becomes anaerobic, allowing different microbes to establish themselves (Lu and Imlay, 2021). As a complex chamber of anaerobic fermentation where dense community of bacteria, archaea, protozoa, and fungi work synthetically to break down plant biomass, *Prevotella* species are reported in studies by Betancur-Murillo et al. (2022), Grabner et al. (2023) and Shinkai et al. (2023), to be notably among the dominant organisms, often comprising up to 20–60% of the total bacterial population due to their remarkable metabolic versatility (Patra and Yu, 2022). Unlike other specialized cellulolytic bacteria, *Prevotella* are non-cellulolytic but highly efficient at degrading a wide range of non-cellulosic polysaccharides, including starch, xylan, and pectin as reported by Jiang et al. (2025). They also play a critical role in nitrogen metabolism (Zhang et al., 2022) by breaking down proteins and peptides into amino acids and ammonia, which are then utilized by other rumen microbes for growth.

Several other bacteria are known to be inhabiting the rumen such as *Ruminococcus albus* (Alam et al., 2024) and *Fibrobacter succinogenes* (Ogawa et al., 2025) that attach directly to plant fibers and reported to possess complex enzyme systems that break down tough cellulose and hemicellulose into simple sugars. Similarly, Adetunji et al. (2022) reported methanogenic archaea represent another important group of microorganisms inhabiting the rumen. They perform a vital “housekeeping” role where they utilize the hydrogen gas produced during fermentation to reduce carbon dioxide into methane. This prevents the accumulation of hydrogen, which would otherwise inhibit fermentation. Similarly, protozoans and fungi existing in the rumen participate actively in regulating bacterial populations through predation (Solomon et al., 2022) and help stabilize pH by sequestering starch as reported by Paswan et al. (2022). This chain of interactions, driven by metabolic exchanges, changes in pH, and oxygen depletion, leads to complex successional patterns. While this process is particularly well-documented in anaerobic environments like the rumen, similar mechanisms of random colonization, environmental selection, and niche modification have been observed in diverse settings, including marine particles (Siebers et al., 2024), pitcher-plant microbiomes (Grothjan and Young, 2022), some foods (Mafe et al., 2025), as well as in controlled lab experiments. The recurrence of these patterns across different ecosystems suggests the possibility of developing general principles to predict how microbial communities assemble in new environments.

To uncover such principles, a shift in perspective is needed, from focusing on pairwise interactions to examining community-wide trophic interactions. Historically, microbiologists have studied microbial relationships by analyzing pairs of species in controlled settings, such as measuring antibiotic effects or co-culturing species in liquid media (Li et al., 2022). This emphasis on pairwise interactions has been partly due to experimental challenges in handling complex communities and the influence of classical ecological models that describe communities in terms of species pairs. However, in highly diverse microbial communities, this approach may not be the most effective way to understand underlying processes.

At first glance, microbial communities may seem too complex to analyse meaningfully or predict how they assemble. However, emerging patterns suggest the existence of universal mechanisms that govern microbial community dynamics (Yang, 2021). This is analogous to the physics of gases, while the behavior of individual gas molecules cannot be predicted; large-scale properties like pressure and volume can be meaningfully described. Similarly, microbial ecology may benefit from a coarse-grained approach, identifying broad principles that govern community assembly. Examining trophic interactions how energy and nutrients flow within these systems could be a key step toward developing a predictive framework for microbial communities. Microbial community dynamics are governed by a set of universal mechanisms that shape the composition, diversity, and function of microorganisms across environments as explained by Shen et al. (2022). One key mechanism postulated was selection, where environmental conditions such as temperature, pH, nutrient availability, and oxygen levels favor the growth of certain microbes over others. This leads to predictable patterns in community structure based on ecological niches (Ji et al., 2024). Another fundamental process highlighted by Ji et al. (2024) was dispersal, the movement of microbes between habitats via air, water, or host organisms. Dispersal connects communities and facilitates the colonization of new environments, thereby influencing microbial diversity and ecosystem resilience (Custer et al., 2022). However, not all microbes that arrive in new environments to them can establish themselves, as they must compete with resident species.

Similarly, drift, or random fluctuations in species abundance, also plays a role, especially in small populations as noted by Ojima et al. (2025). These stochastic changes can lead to the loss or dominance of certain microbes independent of their ecological advantages. It was observed by Arnold et al. (2022) that diversification contributes to microbial dynamics through mutation, horizontal gene transfer, and rapid reproduction and these processes generate genetic variation which enables microbes to adapt quickly to changing conditions and interactions in the ecosystem. Biotic interactions such as competition, mutualism, predation, and cooperation further regulate microbial communities (Iven et al., 2023). For example, microbes may compete for limited resources or form symbiotic relationships that enhance survival. Equally important are some disturbances such as antibiotics (Guan et al., 2025), environmental shifts (Potts et al., 2022), or host immune responses (Wang et al., 2022) can disrupt communities, creating opportunities for new species to invade or previously rare microbes to expand. Together, these universal mechanisms selection, dispersal, drift, diversification, and interaction operate across ecosystems, making microbial community dynamics both predictable in principle and complex in practice.

### 1.4 Microorganisms in One Health

The One Health approach recognizes the interconnectedness of human, animal, and environmental health and highlights the central role of microorganisms and microbiomes in ecosystem functioning, resilience, and overall health across these interconnected systems (Banerjee and van der Heijden, 2022; Ginnan et al., 2025). Microbes play a crucial role in sustaining this balance by influencing soil fertility, plant productivity, animal wellbeing, and human health through complex ecological interactions (van Bruggen et al., 2019). These microbial interactions support sustainable agriculture, enhance ecosystem resilience, and help prevent the emergence and spread of zoonotic diseases by regulating microbial community dynamics across interconnected systems (Banerjee and van der Heijden, 2022).

Microorganisms contribute significantly to ecosystem services by facilitating nutrient cycling, decomposing organic matter, and maintaining soil structure and fertility (Wang et al., 2024). In soils, bacteria and fungi promote plant growth through mechanisms such as biological nitrogen fixation, phosphorus solubilisation, and the production of plant growth–promoting compounds (Timofeeva et al., 2023; Rakhmatova et al., 2026). In addition, symbiotic associations, particularly with mycorrhizal fungi, enhance plant water and nutrient uptake and improve resilience to environmental stresses such as drought (Guo et al., 2024). Consequently, a healthy and diverse soil microbiome is fundamental to sustainable food production, ecosystem stability, and environmental conservation (Banerjee and van der Heijden, 2022).

In animal and human health, microbes play both beneficial and pathogenic roles. The gut microbiota in animals and humans is vital for digestion (Ademosun et al., 2025), immune regulation and disease prevention (Patel et al., 2025). However, some environmental and host-associated microbial communities can support the persistence and transmission of zoonotic pathogens, thereby contributing to disease emergence and spread. Avian influenza, migratory birds (Watabe et al., 2004) and emerging zoonoses enteropathogens and *Cryptosporidium* (Nagano et al., 2006) and unusual viral RNA (Rao et al., 2007) in poultry litter used for making mushroom substrate were regarded as potential threats to human health (Rao et al., 2009). Emerging infectious diseases such as COVID-19, avian influenza, and rabies highlight the importance of monitoring microbial populations at the animal-human-environment interface. One Health strategies emphasize surveillance, vaccination, and antimicrobial stewardship to control these threats (Kumar et al., 2025). Microbes also influence environmental health by degrading pollutants (Nivedita et al., 2025), detoxifying heavy metals (Jeyanthi and Govindarajan, 2025), and contributing to climate change mitigation (Anas et al., 2025). Certain microbial species breakdown plastics (Praveen and Mazumder, 2025), and chemical contaminants (Wu et al., 2025), making them valuable in bioremediation efforts.

Moreover, methane-oxidizing bacteria help reduce greenhouse gas emissions, playing a role in global climate stability (Li et al., 2025). To harness the benefits of microbes while mitigating risks, interdisciplinary collaboration between microbiologists, veterinarians, ecologists, and policymakers is essential. However, Catalano et al. (2024) emphasizes the critical role of whole-genome sequencing in revolutionizing infectious disease surveillance. For it will enable near real-time detection of pathogens and tracking of infection spread, which is vital for public health interventions. However, high-throughput sequencing technologies remain underutilized in many low- and middle-income countries, thereby limiting global capacity for the effective surveillance and study of zoonotic diseases. This technological gap constrains the detection, monitoring, and characterization of emerging pathogens and antimicrobial resistance determinants. Consequently, strengthening genomic infrastructure and bioinformatics capacity in these regions is essential for improving global health preparedness. Within this context, the One Health approach provides a comprehensive framework for integrating microbial research into public health policy, agricultural practices, and environmental conservation efforts, thereby enhancing disease prevention, surveillance, and ecosystem resilience.

### 1.5 Antimicrobial resistance and One Health integration

Antimicrobial resistance (AMR) is a global health issue that most exemplifies the One Health concept (Al-Khalaifah et al., 2025). The One Health approach is characterized as an interdisciplinary collaboration that aims to address issues related to human, animal, and environmental health. Overtime, careless overuse and abuse of antibiotics in humans, animal rearing, and agriculture have turned out to be a threat to population health and existence (Salam et al., 2023).

Resistance can spread to other bacteria in the same or other genus by acquiring mobile genetic elements and resistance genes under the pressure of antimicrobial selection (Deori et al., 2024). Perhaps the most important battlefield for AMR is the human-animal interface. Although antibiotics are widely used in both livestock production and human medicine, they are often administered not only for treatment but also for disease prevention and management, creating strong selection pressures that favor antimicrobial-resistant bacteria. Because of the intense selection pressure this produces, resistant types of bacteria like *Salmonella* or *E. coli* can proliferate at a very fast rate (Vats et al., 2022) via different channels such as direct touch (Kushwaha et al., 2023), food chain (Sagar et al., 2023), or water source contamination (Larson et al., 2023), these “superbugs” spread to people.

The environmental component of the One Health framework also plays a major role in the emergence and dissemination of AMR. Antibiotics, resistant bacteria, and resistance genes can enter soils, water bodies, agricultural slurry, hospital waste streams, and industrial effluents, creating conditions that favor the maintenance and exchange of resistance determinants (Klümper et al., 2024; Abosse et al., 2024; Khan et al., 2022). Environmental reservoirs for the selection and exchange of antimicrobial resistance genes. Once these genes are in the environmental pool, viruses may acquire them and eventually infect humans or wildlife, resulting in an endless cycle of resistance.

The rapid emergence of multidrug-resistant bacteria is particularly concerning given the limited availability of new antimicrobial agents. Of the 32 antibacterial agents in clinical development in 2019, only six were classified as novel by the World Health Organization, Food and Agriculture Organization of the United Nations, United Nations Environment Programme, and World Organisation for Animal Health (2024), underscoring the challenges associated with replenishing the antimicrobial pipeline. This shows how global health systems are being impacted by the shortage of antimicrobials. Similarly, several studies are showing how antimicrobials are becoming less effective against infections caused by antimicrobial-resistant microbes (Zhu et al., 2022), which makes treating these infections challenging and increases both morbidity and mortality rates in the population. To control illnesses brought on by the primary pathogens listed by the WHO, new antimicrobials are required. It is pertinent to note that AMR is a worldwide public health issue for which epidemiological surveillance methods have been developed with the goal of fostering partnerships focused on the health of people and animals as well as the ecosystem's equilibrium (Bertagnolio et al., 2023).

Numerous global institutions, including the World Organization for Animal Health, WHO, and the Food and Agriculture Organization of the United Nations have collaborated to create a Global Action Plan on Antimicrobial Resistance (World Health Organization, 2024). The One Health strategy is completely included in these international initiatives to combat AMR. The conflicting interests of various economic sectors and organizations involved in animal, human, and environmental health are among the several challenges to be solved. The optimal methods for tracking antimicrobial resistance (AMR) and controlling infections, as well as the regulations that should limit the use of antibiotics, must be agreed upon by these parties. As observed by Paramasivam et al. (2023), AMR frequently raises the cost, incidence, and severity of infection while at the same time decreasing the efficacy of antimicrobial therapy. Currently, evidence suggested that the veterinary industry's careless use of antibiotics has resulted in the emergence of several antibiotic-resistant bacteria that are prevalent among human population (Tiamiyu et al., 2024). These include various strains of *Enterococcus* (Caneschi et al., 2023), *Salmonella* and *E. coli* (Sana et al., 2025).

### 1.6 Metagenome-assembled genomes (MAGs) in microbial diversity and ecological functions

Advances in metagenomic sequencing have transformed microbial ecology by enabling the culture-independent investigation of microbial communities. Within these approaches, metagenome-assembled genome (MAG) reconstruction provides genome-level resolution through the recovery of microbial genomes directly from environmental samples. Together, these methods have expanded our understanding of previously uncultured microorganisms and their contributions to ecosystem processes. MAGs have yielded important insights into microbial diversity (Meziti et al., 2021), evolutionary relationships (Xu et al., 2024), and ecological functions across diverse habitats, including oceans (Royo-Llonch et al., 2021), soils (Kazarina et al., 2025), and the human gut microbiome (Zeng et al., 2022). One major contribution of MAGs is their ability to uncover previously unknown microbial lineages. Many microbes cannot be cultured in laboratories, limiting their study. By assembling genomes from metagenomic data, scientists have identified novel bacterial and archaeal phyla, expanding the tree of life. These discoveries reshape our understanding of microbial evolution and their metabolic potential. MAGs have also enhanced our knowledge of microbial metabolic functions, interactions (Setubal, 2021) and antimicrobial resistance (Abdulkadir et al., 2024).

By reconstructing near-complete genomes, researchers can infer the metabolic potential of microorganisms, including pathways involved in carbon and nitrogen cycling, methane production, and sulfur metabolism (Kumar et al., 2023). This has important implications for climate change research, wastewater treatment, and sustainable agriculture. Furthermore, MAGs provide insights into microbial community ecology and can be used to infer potential interactions among microorganisms, including symbiotic and syntrophic associations based on complementary metabolic capabilities. In both clinical and environmental sciences, MAGs are increasingly used for pathogen surveillance, antimicrobial resistance monitoring, and the characterization of microbial populations associated with health and disease. They also facilitate the identification of microorganisms with the potential to degrade pollutants and contribute to ecosystem restoration, thereby supporting bioremediation efforts (Anju et al., 2024; Abdulkadir et al., 2024).

Genome-resolved metagenomic approaches have transformed microbial research by providing genome-level insights into diverse microbial communities, including those involved in pathogen transmission (Abdulkadir et al., 2024; Kim N. et al., 2024). Unlike traditional culture-based techniques, which capture only a small fraction of microbial life, MAGs reconstruct microbial genomes directly from environmental samples (Alteio et al., 2020). This has enabled researchers to study uncultured microorganisms and their roles in disease dynamics, revealing critical interactions that influence pathogen emergence and spread. Metagenomic sequencing and MAG reconstruction facilitate the discovery and lineages that were previously undetectable (Sánchez-Navarro et al., 2022). These discoveries provide insights into microbial evolution and their functional roles in various ecosystems, including soil, oceans, and host-associated microbiomes. Many of these newly identified microbes are linked to host health, influencing disease emergence and pathogen dynamics.

In the context of pathogen transmission, MAGs have been instrumental in identifying microbial communities that harbor or facilitate the spread of infectious agents (Ko et al., 2022). Studies have shown that certain environmental microbes act as reservoirs or vectors for pathogens, affecting transmission dynamics (Bernardo-Cravo et al., 2020). For example, MAGs have uncovered microbial interactions within the gut microbiome, revealing how certain bacteria enhance or suppress pathogenic infections (Ravi et al., 2019). Additionally, environmental surveillance using MAGs has detected potential zoonotic pathogens in wildlife, water sources, and food systems, aiding in outbreak prediction and prevention (Suminda et al., 2022). Similarly, studies by Houf et al. (2009) and Rovetto et al. (2017) showed how *Arcobacter* species is implicated in pathogenicity and public health.

Both emphasize *Arcobacter* as an emerging pathogen, particularly in foodborne illnesses. These findings further contribute to epidemiological and clinical contexts, contributing to food safety and disease monitoring and antimicrobial resistance (AMR) genes of significance to food quality and safety (Nelson et al., 2019). Similar studies using MAGs have uncovered bacterial species that serve as reservoirs for antibiotic resistance genes, facilitating their spread across different microbial populations (Goodarzi et al., 2022). Additionally, MAGs have identified novel viruses and potential zoonotic agents in wildlife and livestock, aiding in early outbreak detection (Kwok et al., 2020).

Beyond identifying reservoirs, MAGs also illuminate microbial interactions that affect pathogen transmission as reported by Lehman et al. (2024). Certain bacteria within microbial communities can promote or inhibit pathogen colonization by competing for resources or producing antimicrobial compounds (e.g., McIlroy et al., 2020; Toner et al., 2021; Millar et al., 2019; Nelson et al., 2019) and for combating antimicrobial resistance (Millar et al., 2021; Abdulkadir et al., 2024). For instance, MAG-based studies of the human gut microbiome have shown that microbial composition influences susceptibility to infections like *Clostridioides difficile* (Martinez et al., 2022). Similarly, MAGs have been used to examine environmental microbiomes, such as wastewater and hospital surfaces, where microbial interactions impact pathogen survival and spread (Kelly et al., 2023). By providing high-resolution genomic data, MAGs enhance disease surveillance and outbreak prediction. They allow researchers to track the movement of microbial strains across ecosystems, identifying transmission pathways that might otherwise go unnoticed. As a result, MAGs play a crucial role in understanding pathogen ecology and developing strategies to mitigate infectious disease risks in both human and environmental health contexts.

### 1.7 Whole genome sequencing, microorganisms and environment

The use of genomics has now taken center stage in microbial research for the characterization and identification of microorganisms based on their pathogenicity, environmental adaptation, and antimicrobial resistance. A key advantage of “omics” approaches, particularly whole-genome sequencing (WGS), is their ability to identify genetic determinants associated with phenotypic traits, virulence, antimicrobial resistance, physiology, and evolutionary adaptation (Lu et al., 2025). The successful application of WGS in characterization, identification, typing, resistance and detection of virulence genes has so far remained its hallmark (Aminu et al., 2024). The much human genome project triggered a visible revolution of sequencing technologies with optimum output in studying diversities at both human and microbial levels. What aided this breakthrough is the parallel progress recorded in computation technologies and software development resulting in a WGS that read and produce a large amount of data across genomes by utilizing modern computer applications in assembling and organizing sequence reads (Brlek et al., 2024).

It is worth noting that WGS has seen a landmark improvement in process and procedures leading to the historic sequencing of the *Haemophilus influenzae* genome, which is the first free-living organism to be sequenced, and was the most pronounced and advanced level sequencing method ever employed until the late 2000s. Similarly, there is a paradigm-shift evidence from WGS reported data that multidrug-resistant *Mycobacterium abscessus* subspecies massiliense was frequently transmitted between patients with cystic fibrosis, which has prompted reconsideration of how infection spreads and relevant control measures to be used. In addition to studies on individual microorganisms, there are several reported studies of using WGS in the environment, hospital settings to suspected pathogen outbreaks and transmission around the globe. In a 2012 study, researchers from the us National Institutes of Health (NIH), USA used WGS to track and monitor a suspected outbreak of carbapenem-resistant *Klebsiella pneumoniae*, which leads to identifying some patients as the source for the transmission events. The high resolving capacity of WGS has promoted it as the new gold-standard method for identification of microorganisms in their ecosystem, outpacing the known conventional methods. Their resolute precision in providing reliable output makes for strong argument for its application in clinical, public health and environmental microbiology laboratories.

## 2 Microbial niches and pathogen reservoirs

While microbial niche refers to the specific ecological role and environment occupied by a microorganism within an ecosystem (Malard and Guisan, 2023), pathogen reservoirs involve all living and non-living habitat on which a pathogen survive (Narayan et al., 2023). It includes the physical, chemical, and biological conditions that allow a microorganism to survive, grow, reproduce, and interact with other organisms. Most studies reported that microbial niches at the animal-environment interface are critical for predicting, preventing, and controlling zoonotic and environmental diseases (Leal Filho et al., 2025). This interface is where animals, humans, and microbes interact, leading to the emergence and spread of infectious diseases. A pathogen reservoir is any environment, organism, or substance that harbors a pathogen, allowing it to persist, multiply, and potentially spread to new hosts (Zvonareva et al., 2025). Natural environments, particularly soil and water, are widely acknowledged as reservoirs for various plant and human pathogenic bacteria. Several bacterial species have been observed transitioning from their natural habitats to a host-associated pathogenic lifestyle (Mazel et al., 2025). However, in most instances, these natural inhabitants do not exhibit virulence in plant or human hosts.

### 2.1 Animal reservoirs (zoonotic reservoirs)

Many studies have reported how animal reservoirs play an essential role in the transmission and persistence of zoonotic diseases, which are infections that can spread between animals and humans (Tomori and Oluwayelu, 2023; Nayak et al., 2025). Zoonotic reservoirs include a wide range of animals, from domesticated livestock to wildlife that harbor pathogens without necessarily exhibiting clinical signs. These reservoirs serve as significant sources of emerging infectious diseases, making their study essential for public health, veterinary science, and environmental conservation. Wildlife species, particularly bats, rodents, and birds, are recognized as major reservoirs of zoonotic pathogens (Davoust and Laidoudi, 2023; Kemenesi and Kurucz, 2025). Bats, for instance, are known to host viruses like SARS-CoV, Ebola, and rabies without typically exhibiting overt disease symptoms (Narayan et al., 2024). Rodents serve as reservoirs for hantaviruses and leptospirosis, while birds can carry avian influenza viruses (Ibarra-Cerdeña et al., 2024).

Domesticated animals, including cattle, pigs, and poultry, can also serve as reservoirs for bacterial and viral pathogens like *Salmonella, Brucella*, and swine flu. The close interaction between humans and these animals increases the risk of zoonotic spillover events. As reported in Billah and Rahman (2024), *Salmonella* is one of the most found foodborne pathogens in animal-derived foods, especially raw chicken, meat, and eggs, poultry slaughterhouses and fresh produce. The study further identified seafood to be a significant source of the pathogen often linked to raw consumption. Contamination arises from terrestrial pollution and handling practices. Pathogens from animal reservoirs can be transmitted to humans through direct contact, consumption of contaminated animal products, or vector-borne transmission (Rawat et al., 2025). For example, rabies spreads through animal bites, while foodborne illnesses like *E. coli* and *Campylobacter* infections occur through contaminated meat or dairy products. Climate change, deforestation, and wildlife trade contribute to increased human-animal interactions, leading to the emergence of new zoonotic diseases, such as COVID-19 (Al Noman et al., 2024).

### 2.2 Human reservoirs

Humans serve as reservoirs for many common infectious diseases (Wilson, 2001). Several respiratory pathogens, as well as illnesses like measles, mumps, streptococcal infections, and certain sexually transmitted diseases, can spread directly between individuals without requiring an intermediary host (Gusain et al., 2024). In many infectious disease cycles, humans act as the primary or sole reservoir, meaning the pathogen relies on human hosts for survival and spread. These pathogens include viruses and bacteria responsible for respiratory infections such as influenza, measles, mumps, and streptococcal pharyngitis, as well as various sexually transmitted infections like gonorrhea, syphilis, and chlamydia. Because these pathogens can pass easily from person to person through droplets, contact, or bodily fluids, the human reservoir plays a crucial role in sustaining disease transmission within populations. Similarly, humans can serve as symptomatic or asymptomatic carriers of disease (Jamrozik and Selgelid, 2019). Some individuals may never exhibit symptoms yet continue to harbor and transmit the pathogen, these are known as asymptomatic or healthy carriers. Others may spread pathogens during the incubation period before symptoms appear (incubatory carriers), after recovery (convalescent carriers), or even for years after infection (chronic carriers), as seen in diseases like typhoid fever and hepatitis B. The eradication of smallpox provides a key example of the importance of human reservoirs. Because humans were the only known host for the virus, global vaccination and isolation strategies were able to interrupt transmission, leading to the disease's elimination.

### 2.3 Environmental reservoirs

Freshwater habitats make up only a small fraction of the Earth's total water volume, yet they play a crucial role in supporting diverse species and contributing to human wellbeing. Lakes and ponds, often referred to as “lentic jewels” of the landscape, provide unique ecological, economic, and cultural benefits (Kim M. K. et al., 2024). With their calm waters and distinct stratification, these habitats serve as vital biodiversity hotspots and genetic reservoirs, hosting numerous endemic species (Bocanegra-García et al., 2023). The biodiversity found in lakes and ponds also delivers essential ecosystem services. Beyond their ecological importance to pathogens, these freshwater systems are essential to agriculture, serving as irrigation reservoirs that help sustain food production for large populations (Baggio et al., 2021). However, these ecosystems face growing threats, with degradation affecting both the species that inhabit them and the ecosystem services they provide. Recognizing the importance of lakes and ponds within the broader freshwater system is essential for their conservation. The intricate web of life in these habitats, particularly their microbial communities, plays a fundamental role in maintaining ecological balance, driving nutrient cycles, and ensuring overall ecosystem health (Srivastava et al., 2023). These microbial populations, mainly composed of bacteria, are crucial to biogeochemical processes, highlighting their indispensable ecological function.

The relationship between freshwater habitats and nearby terrestrial ecosystems, especially vegetation, is particularly significant. Plants and trees influence microbial communities by releasing organic matter through their roots and leaf litter, supplying essential nutrients that support microbial growth and metabolism (Martins et al., 2021). This ongoing nutrient exchange strengthens ecosystem efficiency and health. Additionally, vegetation around lakes and ponds serves as a protective barrier, reducing the entry of pollutants and sediments into the water (Wang et al., 2024). Microbial communities contribute to water purification by breaking down pollutants and absorbing excess nutrients, thereby preventing eutrophication and harmful algal blooms (Lan et al., 2024). Their metabolic flexibility enables them to perform key ecological functions, such as carbon sequestration and nitrogen fixation (Alleman and Peters, 2023). However, human activities, including deforestation, urbanization, and pollution, can disrupt these delicate ecological interactions, altering microbial community composition and function (Aryal et al., 2022). The loss of vegetation reduces organic inputs that sustain the microbiome, weakening the symbiotic relationships that support ecosystem stability. The overall health and sustainability of lakes and ponds depend on both microbial populations and the surrounding vegetation that nourishes them. As research continues to uncover the complexities of these interconnections, it becomes increasingly clear that preserving both terrestrial and aquatic ecosystems is essential for maintaining planetary health (Kim M. K. et al., 2024).

## 3 Environmental factors and pathogen survival in microbial niche

Microbial niches provide the conditions necessary for microorganisms, including pathogens, to survive, multiply, and interact within an ecosystem. The survival and persistence of pathogens in these niches are strongly influenced by environmental factors strongly influence microbial survival, persistence, and community dynamics (Nguyen et al., 2021). These pathogens can persist in open environments, with their survival largely dependent on their ability to utilize nutrients and adhere to surfaces. Several human and animal pathogens have been reported in agricultural soils and plant rhizospheres (Yang et al., 2024), manure (Doddabematti Prakash et al., 2024), and irrigation water (Osman et al., 2024).

Additionally, they can colonize the interior of plants, making them challenging to eliminate through washing or disinfection. These organisms can enter the food chain through splashes of water during rainfall or irrigation, further contaminating food during processing and packaging. In a study by Estrada-Peña et al. (2014), environmental changes significantly influence the distribution of zoonotic pathogens. These changes can alter the habitats and behaviors of both hosts and vectors, which in turn affect the transmission dynamics of diseases to humans. This primarily affect pathogens' persistence and impact on how long they survive outside their hosts, which is crucial for understanding disease spread. For instance, animal agriculture grassland farms in Britain and Ireland, bovine and pig slurry waste spreading are quite common as a natural means of nutrient management. Prevalence of bacterial fecal pathogens in separated and unseparated stored pig slurry has also been monitored (Watabe et al., 2003) and other occurrences of enteric pathogens (e.g., *Cryptosporidium*) in lettuce (Snelling et al., 2007a,b; Moore et al., 2007; Xiao et al., 2006; Shigematsu et al., 2007). Airborne enteric pathogens in aerosol particulates were detectable via molecular identification (Murayama et al., 2010).

Furthermore, such enteric pathogens in farmland soil may also be waterborne and the spread of potential antimicrobial resistance in nearby watercourses, rivers and streams (Moore et al., 2010). AMR resistance could serve as an indicator for monitoring environmental changes to antimicrobial resistance in animal farms, water interface environments and in rare but extreme events such as environmental radiation discharge events (Nakanishi et al., 2012) could reveal if such environmental bacteria of animal and human wellbeing become antibiotic susceptible, impacting One Health perspectives.

### 3.1 Impact of temperature on pathogen survival

All living organisms experience environmental fluctuations, including changes in temperature. The ability to detect and react to temperature variations is widespread, spanning from bacteria to mammals. For many microorganisms, including viruses, archaea, bacteria, fungi, and parasites, temperature is a vital cue that influences growth, development, and pathogenicity (Roncarati et al., 2024). These microbes encounter temperature shifts due to seasonal changes, global warming, interactions with different hosts, and fever responses in infected hosts. In response to temperature fluctuations, whether an increase or a decrease, cells adjust by altering gene expression, producing protective proteins, and slowing down various cellular activities (Lee et al., 2024). This adaptation, known as the heat-shock response, was first identified in 1962 by Ritossa in *Drosophila melanogaster* (Singh et al., 2024).

It is an evolutionarily conserved mechanism characterized by the upregulation of specific proteins, primarily chaperones and proteases, collectively referred to as heat shock proteins (HSPs). These proteins play a crucial role in maintaining protein homeostasis and protecting cells from stress-induced damage. In bacterial pathogens, HSPs contribute not only to protein folding and stress adaptation but also to virulence, influencing host colonization, survival, and the infection process (Figaj, 2025).

Several pathogens utilize the major chaperones GroEL and DnaK as surface-exposed adhesins, facilitating interactions with host cells. For instance, *Mycoplasma pneumoniae*, a respiratory pathogen, employs GroEL and DnaK to bind to human A549 cells and various host proteins, including plasminogen, vitronectin, fibronectin, fibrinogen, lactoferrin, and laminin (Xiu et al., 2024). This suggests their potential role in adhesion and bacterial dissemination during infection. Similar mechanisms have been observed in other bacterial species, such as *Salmonella enterica, Neisseria meningitidis*, and *Listeria monocytogenes* (Ishikawa et al., 2024). Moreover, these chaperones have been found to activate specific signaling pathways in host cells, including the induction of pro-inflammatory cytokine production and the promotion of apoptosis (Chen et al., 2024). The role of temperature-regulated HSPs in virulence extends beyond GroEL and DnaK, as reported in *Francisella tularensis*, a highly infectious intracellular pathogen. The *ClpB* protein, belonging to the Hsp100/Clp family has dual functions of heat-shock survival and ensuring the proper function of the type VI secretion system, a critical component of bacterial virulence (Allsopp and Bernal, 2023). Pathogens' ability to swiftly adapt to temperature changes depends on specialized heat-sensing systems that detect environmental cues and initiate appropriate response pathways (Roncarati et al., 2024).

### 3.2 pH (acidity and alkalinity), moisture and water activity

Microorganisms can thrive in diverse environments, including those with extreme pH levels (Shu and Huang, 2022). Certain species of Bacteria and Archaea can survive in highly acidic environments, such as acid mine drainage systems with a pH of 3 or lower (Du et al., 2022). However, most microbes are neutrophiles, preferring pH conditions between 5.5 and 8.0. Extreme pH levels typically denature proteins, disrupting cellular metabolism and threatening viability. However, microorganisms surviving in such conditions have evolved mechanisms to regulate their internal pH and maintain homeostasis (Wani et al., 2022). Acidophiles, for instance, have developed protective adaptations to shield their cell membranes from acidic damage (Chen, 2021). Some create biofilms to slow the entry of harmful molecules, while others secrete buffering agents to neutralize acidity in their immediate environment. Additionally, some acidophiles possess H^+^ pumps that actively expel protons, ensuring that their internal pH remains close to neutral (Chen, 2021).

The inability of foodborne pathogens to proliferate in environments with low water activity (aw) does not eliminate the risk of foodborne illness (Morasi et al., 2022). For instance, *Salmonella* and Shiga toxin-producing *Escherichia coli* (STEC), associated with foodborne illness, have low infectious doses in products with low water activity (Walker et al., 2024). Once desiccated, *Salmonella* can persist in dry environments, even in the presence of antimicrobial substances like oregano and clove, for extended periods (De Almeida et al., 2023). This pathogen is known for its resilience in dry conditions and poses a challenge in acidic, low-moisture foods (Chaggar et al., 2024). For a pathogen to survive in a low-moisture environment, regardless of pH, it must first withstand desiccation. Most studies on desiccation and survival in acidic conditions have not directly measured pH but have instead focused on the type of acid used or acidic pretreatments. For example, a study by Deliephan et al. (2023) examined *Salmonella* survival after treatment with 2-hydroxy-4-methylbutyric acid applied as a liquid and as a dry powder to pet food kibble.

Although survival was reduced in both cases, the pH and aw of the kibble before and after treatment were not reported, making it unclear how pH influenced survival. Several researchers have investigated the effects of pre-treating fruit with ascorbic acid or citric acid before drying on the survival of *Salmonella* and *E. coli* (Suwannate, 2022; Zhang et al., 2024). Lower pH resulting from acid treatment generally led to greater reductions in bacterial populations as reported in most of these studies. However, the effect of pH itself was difficult to isolate due to differences in food composition. These studies also involved cultures that were adapted to acidic conditions before use in acidic products but were not specifically adapted for desiccation resistance. While prior exposure to acidic conditions may provide some level of cross-protection against other stressors, the extent to which this adaptation confers resistance to desiccation or low-moisture environments remains unclear.

## 4 Host–microbe interactions

Host–microbe interactions encompass the diverse relationships between microorganisms and their hosts, ranging from mutualism and commensalism to parasitism and disease (Casadevall and Pirofski, 2000; Gestal et al., 2024). These interactions are dynamic and context-dependent, with their outcomes influenced by host characteristics, microbial traits, and environmental conditions. Although microorganisms have historically been classified according to their disease-causing potential, it is now recognized that many host-associated microbes can exist without causing disease and may contribute positively to host health and ecosystem functioning.

The distinction between commensal, opportunistic, and pathogenic microorganisms is often fluid. Under favorable conditions, many microbes coexist with their hosts without adverse effects, whereas changes in host immunity, microbial community composition, or environmental stress can facilitate disease development (Sen et al., 2016). Similarly, microbial persistence and asymptomatic carriage can contribute to pathogen maintenance and transmission within populations, as demonstrated for pathogens such as *Salmonella enterica* serovar Typhi (Hoffman et al., 2023; La Rosa et al., 2022). Understanding these interactions is therefore essential for explaining how microbial communities influence host health, pathogen transmission, and disease dynamics within the One Health framework.

### 4.1 How host behavior and immunity can affect disease transmission

Host behavior and immune function are major determinants of pathogen transmission within and across ecosystems. Behavior influences contact rates among hosts and between hosts and environmental reservoirs, whereas immune function determines susceptibility to infection, pathogen shedding, and infectiousness. Social behaviors such as grooming, migration, communal nesting, and feeding can facilitate microbial exchange and influence disease transmission dynamics (Balasubramaniam et al., 2019; Plowright et al., 2017). High-density livestock production systems and migratory wildlife populations further demonstrate how host behavior can promote pathogen dissemination across local and global scales (Arruda et al., 2018; Xu et al., 2016).

Host immunity plays a central role in maintaining microbial homeostasis by limiting pathogen proliferation while preserving beneficial microbial communities. Variations in immune function arising from genetics, nutrition, stress, or prior exposure can generate substantial heterogeneity in disease susceptibility and transmission within populations. For example, immunosuppression may increase pathogen shedding and transmission risk, whereas herd immunity can reduce disease spread by limiting the availability of susceptible hosts (Archie and Theis, 2011; Stein, 2011, 2022). Beneficial microbial communities can also strengthen host immune responses, thereby enhancing resistance to infection (Round and Mazmanian, 2009).

Environmental stressors, including temperature fluctuations, climate change, and pollutant exposure, can alter immune function and host–pathogen interactions, with important consequences for disease emergence and transmission. Such interactions highlight the interconnected roles of host behavior, immunity, and environmental conditions in shaping pathogen dynamics across One Health systems (Estrada-Peña et al., 2014; Rollins-Smith, 2017; Carpi et al., 2015).

### 4.2 The role of host microbiota in preventing or facilitating pathogen invasion

The host microbiota functions as both a barrier and a facilitator of pathogen invasion. Under normal conditions, resident microbial communities protect hosts through competitive exclusion, production of antimicrobial compounds, and modulation of immune responses (Pickard et al., 2017; Billah and Rahman, 2024). In humans and animals, commensal microorganisms such as *Lactobacillus* and *Bifidobacterium* can inhibit pathogen colonization by competing for nutrients and attachment sites, whereas disruptions of microbial communities (dysbiosis) can increase susceptibility to infection (Round and Mazmanian, 2009; Buffie et al., 2012; Spigaglia, 2024).

In plant systems, beneficial rhizosphere microorganisms, including plant growth-promoting rhizobacteria and mycorrhizal fungi, contribute to pathogen suppression and improved host resilience through nutrient acquisition, competitive exclusion, and modulation of plant defense responses (Berendsen et al., 2012).

Beyond direct competition, microbiota members can inhibit pathogen establishment through the production of bacteriocins, short-chain fatty acids, and quorum-sensing inhibitors that reduce pathogen growth and virulence (Liévin-Le Moal and Servin, 2014; Singh and Bhatia, 2021). However, microbial communities may also facilitate pathogen persistence under certain ecological conditions. Dysbiosis, cross-feeding interactions, and horizontal transfer of antimicrobial resistance and virulence genes can enhance pathogen survival and transmission. The soil environment is particularly important as a reservoir of resistance determinants that may eventually reach clinically relevant pathogens (Forsberg et al., 2012; Abdulkadir et al., 2024).

The balance between protective and facilitative microbial interactions is therefore a key determinant of disease outcomes across human, animal, and plant systems. Maintaining diverse and resilient microbiomes is increasingly recognized as an important component of disease prevention and antimicrobial resistance mitigation within the One Health framework (Rovetto et al., 2017).

### 4.3 Targeted interventions to reduce pathogen persistence and transmission

Understanding microbial niches can support the development of targeted interventions to reduce pathogen persistence and transmission across human, animal, and environmental systems. Within a One Health framework, effective strategies include integrated disease surveillance, antimicrobial stewardship, environmental hygiene, improved biosecurity, and monitoring of high-risk interfaces where pathogen spillover is likely to occur (Kaiser et al., 2022; Vora et al., 2023). Ecological drivers of disease emergence, including habitat degradation, biodiversity loss, wildlife–livestock interactions, and climate change, should also be considered in environmental management and public health policies (Plowright et al., 2024). By addressing the environmental conditions that facilitate pathogen survival, establishment, and transmission within microbial niches, these interventions can complement conventional medical and veterinary approaches and contribute to the prevention of emerging infectious diseases and antimicrobial resistance.

## 5 Conclusion

Microbial niches are fundamental determinants of microbial distribution, community assembly, and host-pathogen interactions across interconnected human, animal, and environmental systems. As highlighted throughout this review, the structure and function of microbial communities are shaped by complex interactions among environmental conditions, host behavior, immune responses, and microbial ecological processes. These interactions influence pathogen persistence, antimicrobial resistance dissemination, and disease emergence, underscoring the importance of microbial ecology within the One Health framework.

Advances in metagenomics, genome-resolved analyses, and multi-omics technologies have substantially improved our ability to characterize microbial communities and investigate the ecological mechanisms underlying pathogen transmission and antimicrobial resistance. Metagenomics and Metagenome-assembled genomes (MAGs) have provided genome-level insights into previously uncultured microorganisms, expanding our understanding of microbial diversity, environmental reservoirs, and functional capabilities across diverse ecosystems.

Despite these advances, important knowledge gaps remain regarding the mechanisms by which microbial interactions, environmental change, and host-associated factors influence pathogen emergence and persistence. Future research should integrate metagenomics with ecological theory, host immune profiling, and environmental surveillance to develop predictive frameworks for disease risk assessment and intervention. Such efforts are especially important in under-resourced regions, where limited surveillance capacity may hinder the early detection of emerging infectious diseases and antimicrobial resistance threats.

A deeper understanding of microbial niches across the One Health continuum will strengthen our capacity to predict, monitor, and mitigate disease transmission while supporting ecosystem resilience and sustainable health outcomes. Continued investment in interdisciplinary research, surveillance infrastructure, and genome-resolved microbial studies will be essential for advancing evidence-based One Health strategies in an increasingly interconnected and rapidly changing world.
